# Supplementary material for: Identification of photoperiod-induced specific miRNAs in the adrenal glands of Sunite sheep (Ovis aries)
Source: Front Vet Sci. 2022 Jul 22;9:888207. doi: 10.3389/fvets.2022.888207 (PMC9354845; doi:10.3389/fvets.2022.888207)
Supplement: Supplementary file 1 [file Data_Sheet_1.ZIP › Supplementary file/miRDeep2 file.zip!/miRDeep2 file/Know_Structure/SPLP42A1/oar-mir-3957.pdf]

miRBase precursor : oar-mir-3957
Total read count : 102
oar-miR-3957-5p read count : 81
oar-miR-3957-3p read count : 20
remaining reads : 1

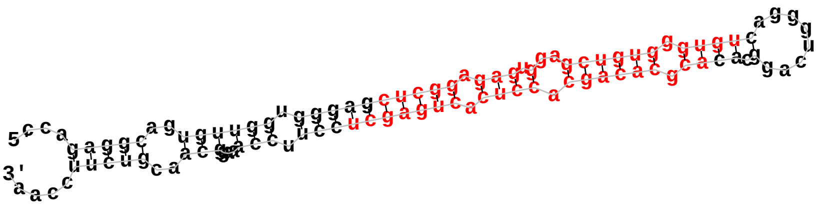

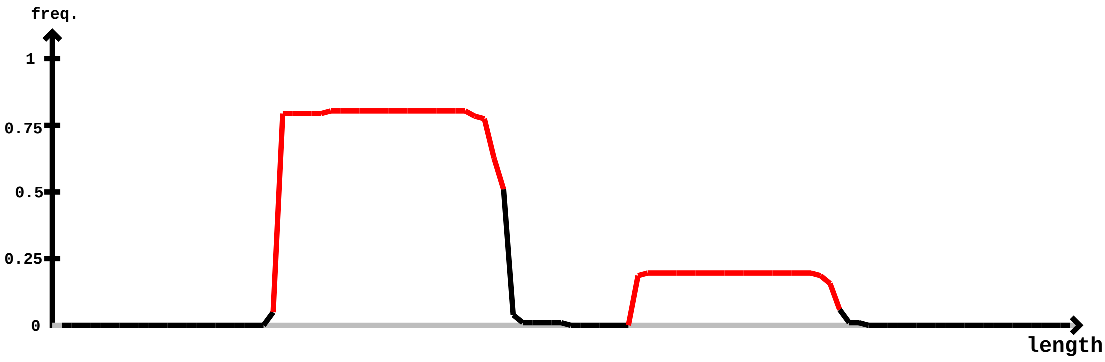


```
                      oar-miR-3957-5p
                                                          oar-miR-3957-3p
5'- ccagaggcaguguuggugggagcucggagaguggagcugugggugucagggucaggcacacgcacagcaccucacugagcuccuuccaaaggcaacgucuuccaa   -3'    exp
    ...(((((..((((((.((((((((((.(((.(..((((((.(((((........).)))).)))))).)))).)))))))))).)))...)))..)))))....    reads      mm         sample
    .....................gcucggagaguggagcugugggug............................................................    2          0          seq
    .....................gcucggagaguggagcugugggugu...........................................................    3          0          seq
    ......................cucggagaguggagcugugg...............................................................    2          0          seq
    ......................cucggagaguggagcuguggg..............................................................    1          0          seq
    ......................cucggagaguggagcugugggu.............................................................    15         0          seq
    ......................cucggagaguggagcugugggug............................................................    10         0          seq
    ......................cucggagaguggagcugugggugu...........................................................    45         0          seq
    ......................cucggagaguggagcugugggugucc.........................................................    3          0          seq
    ...........................agaguggagcugugggugucagggu.....................................................    1          0          seq
    ...........................................................acgcacagcaccucacuga...........................    1          0          seq
    ...........................................................acgcacagcaccucacugag..........................    3          0          seq
    ...........................................................acgcacagcaccucacugagc.........................    9          0          seq
    ...........................................................acgcacagcaccucacugagcu........................    5          0          seq
    ...........................................................acgcacagcaccucacugagcucc......................    1          0          seq
    ............................................................cgcacagcaccucacugagc.........................    1          0          seq
```
